# Bone Health Impairment in Patients with Hemoglobinopathies: From Biological Bases to New Possible Therapeutic Strategies

**DOI:** 10.3390/ijms25052902

**Published:** 2024-03-01

**Authors:** Alessandra Di Paola, Maria Maddalena Marrapodi, Martina Di Martino, Giulia Giliberti, Giuseppe Di Feo, Deeksha Rana, Shakeel Ahmed, Maura Argenziano, Francesca Rossi, Domenico Roberti

**Affiliations:** 1 Department of Woman, Child and General and Specialist Surgery, University of Campania “Luigi Vanvitelli”, 80138 Naples, Italy; alessandra.dipaola@unicampania.it (A.D.P.); mariamaddalena.marrapodi@unicampania.it (M.M.M.); martina.dimartino@policliniconapoli.it (M.D.M.); giuseppe.difeo.bio@gmail.com (G.D.F.); maura.argenziano@unicampania.it (M.A.); domenico.roberti@unicampania.it (D.R.); 2 Department of Experimental Medicine, University of Campania “Luigi Vanvitelli”, 80138 Naples, Italy; giulia.giliberti@unicampania.it (G.G.); deeksha.rana@unicampania.it (D.R.); shakeel.ahmed@unicampania.it (S.A.)

**Keywords:** hemoglobinopathies, thalassemia, sickle cell disease (SCD), alteration of bone metabolism, osteoporosis (OP)

## Abstract

Hemoglobinopathies are monogenic disorders affecting hemoglobin synthesis. Thalassemia and sickle cell disease (SCD) are considered the two major hemoglobinopathies. Thalassemia is a genetic disorder and one of the major hemoglobinopathies determined by an impairment of globin chain production, which causes an alteration of erythropoiesis, an improvement in hemolysis, and an alteration of iron homoeostasis. In SCD, the mutations are on the β-globin chain of hemoglobin which results in a substitution of glutamic acid by valine with consequent formation of Hemoglobin S (HbS). Several factors are involved in bone metabolism alteration in patients with hemoglobinopathies, among them hormonal deficiency, bone marrow hyperplasia, iron overload, inflammation, and increased bone turnover. Bone metabolism is the result of balance maintenance between bone deposition and bone resorption, by osteoblasts (OBs) and osteoclasts (OCs). An impairment of this balance is responsible for the onset of bone diseases, such as osteoporosis (OP). Therefore, here we will discuss the alteration of bone metabolism in patients with hemoglobinopathies and the possible therapeutic strategies to contain and/or counteract bone health impairment in these patients, taking into consideration not only the pharmacological treatments already used in the clinical armamentarium, but also the new possible therapeutic strategies.

## 1. Introduction

Hemoglobinopathies are the most common monogenic diseases worldwide which are characterized by altered hemoglobin synthesis [1]. Hemoglobin is a tetrameric protein present in erythrocytes involved in oxygen transport in the human body. It is constituted of four globins, which are coded by globin genes. DNA variants in these genes are responsible for the onset of hemoglobinopathies. Hemoglobinopathies can be determined by an alteration of α- or β-globin synthesis (determining the onset of α- and β-thalassemia syndromes, respectively), or by hemoglobin structural impairment (resulting in the onset of several diseases such as sickle cell disease (SCD), hemolytic anemia, polycythemia, or erythrocytosis) [1,2,3].

Thalassemia is a genetic disorder and one of the major hemoglobinopathies determined by an impairment of globin chain production, which causes an alteration of erythropoiesis, an improvement in hemolysis, and an alteration of iron homoeostasis. Thalassemic patients exhibit multifactorial clinical manifestations, or any complications or extraordinarily strong consequences which lead to the necessity of transfusions [4]. β-thalassemia is distinguished by minor, major, or intermedia types based on the impairment of α-globin or β-globin chain equilibrium, the grade of anemia, and the clinical manifestation at diagnosis [5]. Heterozygous mutation of β-globin gene leads to the onset of β-thalassemia minor, which is not responsible for severe clinical manifestations: patients exhibit clinically asymptomatic-microcytic anemia or any identified hematological abnormalities (silent carriers). Conversely, homozygous or compound heterozygous mutations in the β-globin gene cause major or intermedia β-thalassemia [5]. β-thalassemia major (TM) determines severe anemia in childhood and leads patients to lifelong transfusion dependency, while β-thalassemia intermedia can appear later in life causing mild to moderate anemia and different transfusion requirements depending on condition [6,7,8].

α-thalassemia is caused by an impairment of synthesis of α-globin chains [9]. It can manifest itself in two different main forms: α+-thalassemia and α0-thalassaemia [5]. α+-thalassemia derives from different point mutations and can be present in several forms [5]. Heterozygous mutations are silent, while homozygous mutations cause mild hypochromic anemia [5]. Particularly, when only one gene is mutated, α-thalassemia is known as “silent” α-thalassemia; conversely, when two genes are affected by mutation, we have an α-thalassemia trait. Compound heterozygotes or homozygotes have HbH disease and show different clinical manifestations, ranging from moderate to severe hemolytic anemia. In particular, when there are prominent levels of β-like globin chains, a non-functional β chain tetramers—called HbH (β4 tetramers)—will appear in adults, and a γ chain tetramers—called Hb Bart’s (γ4 tetramers)—will appear in the fetal period [9]. There is also another form of α-thalassemia, α0-thalassaemia, which is caused by the deletion of both linked α-globin genes: Hb Bart’s Hydrops Fetalis Syndrome [5,9].

SCD is also a major hemoglobinopathy induced by mutations in the β-globin chain of hemoglobin, which results in a substitution of glutamic acid by valine in the sixth position of this chain with consequent formation of Hemoglobin S (HbS) [2]. This disease often leads to chronic hemolytic anemia, severe acute and chronic pain, and end-organ damage, causing premature mortality [2].There are several factors, in particular genetic but also acquired, which are involved in the alteration of bone metabolism and, consequently, in the pathogenesis of osteoporosis (OP) in thalassemia [10]. Bone marrow expansion, endocrine dysfunction, and iron overload contribute to the onset of OP in thalassemic patients [11].

OP represents a very frequent (approx. 40–50%) and hard to treat complication in patients affected by hemoglobinopathies [11]. Prevention and early diagnosis are certainly the better strategies to manage this issue and the available anti-osteoporotic therapies are currently good for the scope, even though they are not decisive for patients. They normally experience a very low quality of life and, furthermore, osteopenia and OP are leading causes of morbidity [11,12].

The pathogenesis of OP in thalassemia is multifactorial. There are several risk factors involved in OP development in thalassemic patients, among them, marrow expansion, iron and chelator toxicity, hormonal deficiency, and increased bone turnover [12]. In particular, both anemia itself and iron overload affect bone health. The alteration of erythropoiesis determines an expansion in bone marrow by increasing the number of erythroid precursors. This bone marrow expansion is inhibited by transfusion therapy and the grade of inhibition derives from the transfusion regimen; indeed, in the case of hypertransfusion, erythroid activity is altered. However, multiple factors associated with the alteration of erythropoiesis and anemia can affect and influence bone homeostasis, acting in two different ways: by inhibiting bone formation and promoting bone resorption. Among these factors, we can find increased levels of erythropoietin and decreased levels of hepcidin.

Iron overload is one of the main factors involved in the onset of OP in thalassemia. It is a result not only of the transfusion but also of an impairment of the hepcidin–ferroportin axis, which is mainly involved in the regulation of iron metabolism. When disrupted it causes an increased absorption of iron in the gastrointestinal tract. Iron accumulation is the main responsible factor for OP development in thalassemic patients, determining an alteration in bone deposition and also causing secondary complications (cardiac dysfunction, endocrine disorders, or liver diseases), all involved in OP onset [11].

Although patients with TM are subjected to regular transfusion and consequent iron chelation therapy, osteopenia/OP turns out to be the main responsible factor for morbidity in these patients, determining increased mortality rate as compared with the general population [10,11,13]. TM patients need long-term transfusion therapy in order to ameliorate life expectancy [14,15]. Unfortunately, these transfusions cause a secondary iron overload which is responsible for organ damage and endocrine system impairment [15,16,17]. An increase in iron concentration in bone tissues leads to OP onset [15,18]. Iron accumulation in bone alters its metabolism, by determining a reduction in both mineralization and osteoid maturation thus causing focal osteomalacia [19]. Since there is not a specific mechanism that inhibits iron accumulation in end organs, the use of iron chelators is requested in order to remove excess iron and prevent or reduce the severe consequences of iron overload [15,20,21]. Actually, three different iron chelators are used in clinics: deferoxamine (DFO), deferiprone (DFP), and deferasirox (DFX) [22]. In particular, the oral iron chelator DFX is well tolerated, and it can counteract iron overload derived from transfusion [15]. In 2014, Rossi et al. reported that in TM the excess of iron derived from transfusion altered osteoclasts’ (OCs) activity, determining their hyperactivation, and that after administration of iron chelators, their activity was restored [18].

As already discussed, OP is multifactorial, therefore iron overload does not represent the only cause of the alteration of bone metabolism in thalassemic patients. In particular, a reduction in growth and sex hormone levels represents another risk factor for bone loss in thalassemic patients. The decrease in these hormones levels is a consequence of anemia and its treatment, which determines skeletal structure impairment and marrow expansion [12]. Moreover, the impairment of kidney function also contributes to the alteration of bone metabolism and OP onset. Thalassemic patients sometimes develop renal dysfunction as a result of iron overload or also of chelator toxicity, anemia, oxidative stress, and hypoxia [11,12]. The typical reduction in vitamin D levels in thalassemic patients represents another factor risk for the onset of OP, since this lower concentration is responsible for a reduction in bone mineral density (BMD). Finally, the reduced physical activity is involved in the alteration of BMD in thalassemic patients.

Although levels of both hemoglobin and iron can be restored, impaired bone metabolism with increased bone resorption and consequently reduced BMD is still observed in TM patients [23]. The hyperactivation of OCs depends on an altered receptor–activator of the nuclear factor-kappa B ligand (RANKL)/osteoprotegerin (OPG) signaling [24,25] and on expression of tartrate-resistant acid phosphatase (TRAP) and cathepsin K (CTK) [26]. TRAP and CTK are both enzymes considered as markers of OC activity, involved in bone resorption [27]. TRAP is an iron-phosphoesterase which is activated by proteolytic cleavage in an exposed loop domain by the cysteine proteinase CTK [26,27]. Therefore, they can be considered as therapeutic targets to contain the alteration of OC activity in thalassemic patients.

In order to counteract the bone resorption in TM, it has been proposed to use bisphosphonates (BP), such as alendronate, neridronate, and zoledronate [28]. BPs are well-tolerated resorptive compounds with the ability to reduce bone loss and to increase the BMD, but the number of studies on their use is extremely limited [28,29].

Recently, much interest has been directed to the use of Teriparatide (TP), which is the only anabolic drug approved in Italy for the cure of OP, in particular in postmenopausal, male, and glucocorticoid-induced OP [29]. TP is able to improve the BMD and decrease the risk of fractures [30]. Preliminary case reports demonstrated that TP induced an improvement of BMD in TM patients, leading to the differentiation of bone cells towards the osteoblast (OB) lineage, inhibiting the apoptosis of OBs and osteocytes, and promoting the activation of OBs [31]. Since there are only a few studies about the effects of TPs in TM patients, other future investigations will be needed in order to better highlight their role in counteracting the impairment of BMD in TM patients.

In recent studies, the possibility to use the endocannabinoid/endovanilloid (EC/EV) system as a therapeutic target in bone disease has emerged [26,32,33,34,35]. This system is constituted by the functional transient receptor potential vanilloid type 1 (TRPV1) channel, the cannabinoid receptors type 1 and 2 (CB1/CB2), and all the metabolic enzymes involved in biosynthesis and degradation of endocannabinoids [36]. Rossi et al. demonstrated that OCs express the EC/EV system and that the administration of the selective agonists of the EC/EV receptors can modulate OCs activity and function [26,35]. Moreover, it has been proven that in OCs from TM patients there is an alteration in the expression of TRPV1, CB1, and CB2, with a prevalence of TRPV1 and CB1, which promote OCs activity [26]. The stimulation of TRPV1 with its selective agonist resiniferatoxin (RTX) induces an increase in CB2 receptor expression and a strong reduction in CTK and the number of activated OCs, thus allowing the proposal of TRPV1 as a therapeutic target in counteracting OP in TM patients.

**Figure 1:**
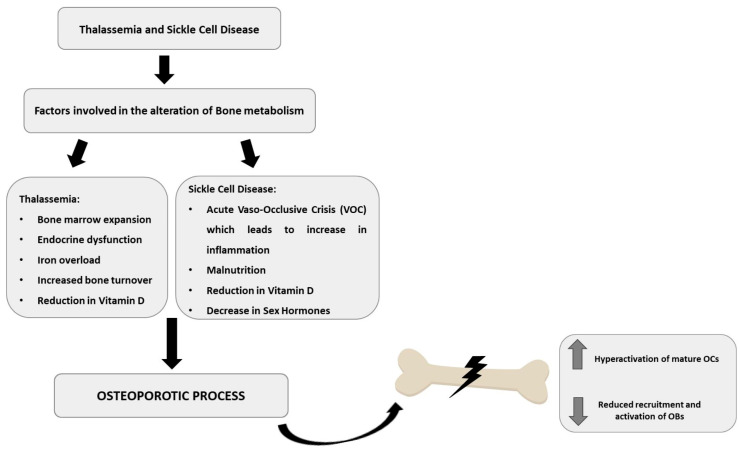
Alteration of bone metabolism in patients with hemoglobinopathies. Patients with hemoglobinopathies, thalassemia (TM) and sickle cell disease (SCD) show an alteration of bone metabolism with hyperactivation of osteoclasts (OCs) and reduced recruitment and activation of osteoblasts (OBs) which leads to an impairment of bone health.

Also, in SCD patients, an impairment of bone health with onset of fractures, chronic pain, and long-term disability is reported [37]. Avascular necrosis and acute vaso-occlusive crisis (VOC) are the main mechanisms involved in the impairment of bone metabolism in SCD [38,39]. In SCD patients, bone health alteration can result in bone marrow necrosis, orbital bone infarction, dental problems, OP, osteopenia, vertebral collapse, increased risk of fractures, bone pain, and osteomyelitis [39]. In SCD, an impairment in the balance between osteoclastogenesis and osteoblastogenesis is also reported, with reduced recruitment and activation of OBs [17,38,39]. Other biological processes are involved in the impairment of bone metabolism in SCD, such as hemolysis, and decreased levels of vitamin D and sex hormones [39,40]. This review offers an overview of the alteration of bone metabolism in patients with hemoglobinopathies and of the possible therapeutic strategies to contain and/or counteract bone health impairment in these patients, taking into consideration not only the pharmacological therapies already known, but also the new possible therapeutic strategies (Figure 1).

## 2. Bone Metabolism: Cells and Pathways Involved in Its Modulation

Bone is a dynamic mineralized connective tissue responsible for different crucial functions in the human body, by giving mechanical support to vital organs and promoting locomotion, determining hormone secretion, representing a storage site for mineral ions (calcium and phosphate), and a niche site for bone marrow [41,42]. It is constituted by organic and inorganic fractions. The organic fraction includes bone cells (OBs, bone lining cells, OCs, and osteocytes), organic matrix (fibrous proteins), osteonectin, osteocalcin, proteoglycans, and glycoproteins. In particular, the organic matrix is the main responsible factor for giving flexibility and strength to bones [41,43]. The inorganic fraction represents 65% of total bone weight, and it is constituted by hydroxyl apatite, which gives hardness and rigidity to bones [41]. Although bone may appear to be an inert tissue, it is highly dynamic, showing a finely regulated metabolism and a balance between the processes of bone matrix deposition by OBs and bone resorption by OCs [43,44].

OBs are cuboidal cells responsible for deposition of bone matrix [44,45]. They derive from mesenchymal stem cells (MSCs) in bone marrow [44,46]. The differentiation of MSCs in OBs is finely regulated by several molecules and pathways, particularly by bone morphogenic proteins (BMPs) and wingless-related integration site (WNT) pathways [46].

When OBs complete their bone matrix deposition activity, they can undergo three different possible processes: apoptosis, transformation in bone lining cells, or differentiation in osteocytes [46,47].

Osteocytes represent a major part of bone cells (90–95%). Their differentiation begins from MSCs that differentiate in OBs and consists of different stages: osteoid-osteocyte, pre-osteocyte, young osteocyte, and mature osteocyte [44,48]. When the formation process of bone is finished, some OBs differentiate in osteocytes which remain embedded in the bone matrix.

Osteocytes exert endocrine properties, by releasing paracrine and endocrine compounds in order to modulate the activity of cells of bone or bone marrow niche and also cells of distant organs [49,50]. In particular, osteocytes have a pivotal role in modulating bone homeostasis, by regulating OBs and OCs activities [49,51]. They are responsible for the release of RANKL and OPG, thus participating in bone resorption, but they also secrete SOST and Dickkopf-related protein 1 (DKK1), thus contributing to bone formation [51,52].

OCs are terminally differentiated multinucleated cells localized on the trabecular surface [53,54]. They derive from mononuclear cells of the hematopoietic stem cell lineage, following stimuli by varied factors. The most important signals for OCs differentiation come from osteoprogenitor mesenchymal cells and OBs which secrete the macrophage colony-stimulating factor (M-CSF), and from OBs, osteocytes, and stromal cells which secrete receptor activator of RANK-L [44,55]. Moreover, there are also inhibitory stimuli for OCs, among them OPG, which is a soluble protein released by stromal cells that binds RANKL, thus not allowing the interaction between RANK and RANK-L. 

A characteristic property of OCs is represented by the presence of lysosomes containing high levels of TRAP and CTKK, main markers of OCs activity [53]. RANK-L is a member of the TNF superfamily of cytokine. Its receptor, RANK, with partial homology to a portion of the extracellular domain of human CD40, a member of TNF receptor superfamily, was previously discovered to be involved in T-cell activation and dendritic-cell function [50].

### 2.1. Bone Remodeling: A Finely Regulated Process

Bone remodeling is a finely regulated process responsible for the continuous activity of bones. It is characterized by the three following phases [42]:

- Bone matrix resorption by OCs;
- Transition phase between bone matrix resorption and deposition, in which the main protagonists are macrophage-like cells;
- Bone matrix deposition by OBs [42].

There is tightly regulated crosstalk between OCs and OBs during bone remodeling: OCs remove old bone matrix and OBs produce new bone matrix, resulting in continuous bone activity [56]. OCs can resorb bone matrix, releasing several compounds, like acid and proteolytic enzymes. Among them, the most important is CTK which is able to cleave proteins of bone matrix. OBs deposit bone matrix by releasing type I collagen, osteocalcin, and alkaline phosphatase [56].

**Figure 2:**
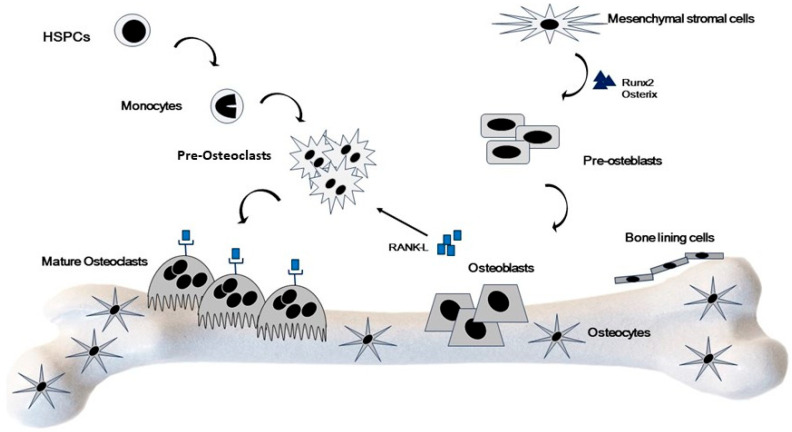
Cellular bone physiology and metabolism. Bone homeostasis is a finely regulated process based on deposition of new bone matrix and bone resorption. This balance is based on the activity of OBs, cells derived from MSCs committed to osteoprogenitors cells and OCs, coming from monocytes/macrophages cell lineage.

In physiological condition, balanced bone remodeling is ensured by two finely regulated processes: osteoclastogenesis and osteoblastogenesis [57]. A key role is played by RANK-L which allows differentiation of OCs precursors (hematopoietic and OB lineage cells) in mature OCs. At the same time, OBs differentiation depends on OC lineage cells of bone microenvironment [57]. Therefore, there is strong crosstalk between these two processes, guaranteed by cell–cell communication between OCs and OBs [56,57] (Figure 2).

As already discussed, the bone remodeling process is a finely regulated process, in which not only OBs and OCs communication plays an important role, but also a lot of released factors are involved in maintaining the equilibrium of this process. There are three main hormones which modulate OCs activity during bone resorption: parathyroid hormone (PTH), estrogen, vitamin D3 [1,25(OH)2 vitamin D3], and calcitonin (CT) [58]. In recent years it has also been reported to have a significant role in regulating bone remodeling by several growth factors (TGF-β, WNTs, IGFs, FGFs, BMPs). PTH is released by the parathyroid glands and is responsible for blood calcium homeostasis and for modulating bone mass. Estrogen mitigates OCs activity, by reducing osteoclastogenesis and stimulating their apoptosis. Vitamin D—particularly its active form—is involved in bone homeostasis. Calcitonin (CT) is released by the thyroid C cells and acts on OCs by influencing their activity [58].

In conclusion, bone is not a static tissue, but is highly dynamic, showing a balance between two processes: bone resorption and bone deposition. This equilibrium is tightly regulated not only by bone cells communication, but also by other signals coming from several hormones. The alteration of bone remodeling is the cause of several bone diseases [43,56,57,58,59].

### 2.2. Bone Metabolism Impairment and Osteoporosis in Some Pathophysiological Situations, the Alteration of the Bone Itself and the Subsequent Impairment of Its Own Function Is Caused by the Dysregulation of Different Molecular Pathways, Involved in the Metabolism of Osteoblasts, Osteoclasts, and Osteocyte, Thus Leading to a Variety of Bone Metabolic Diseases

In particular, the decline of bone formation processes depends both on the reduced recruitment of OBs and on the impaired metabolic activity of mature OBs [60]. However, bone metabolism is not an isolated process. OCs and OBs interact with each other. Under physiological conditions, bone resorption and bone formation maintain a dynamic balance. Considering bone formation, it can be performed either through intramembrane osteogenesis or through cartilaginous osteogenesis [61,62].

Therefore, bone metabolism is one of the most important metabolic processes in the human body. Once its homeostasis is out of balance, it will cause a series of problems, such as the occurrence of bone diseases. A deeper understanding of these regulatory mechanisms is not merely conducive to the prevention of bone diseases, but also conducive to the clinical search for relevant therapeutic targets. OP is a bone disease characterized by a significant reduction in bone mass, inadequate bone growth, and increased fragility of bones, thus resulting in microarchitectural bone impairment [26,60]. It affects more than 200 million people globally and contributes to 1.66 million hip fractures annually [61]. One in three women and one in five men over the age of fifty anticipated suffering an osteoporotic fracture throughout their lifetime, making this a serious risk [62].

There are primary and secondary types of OP. Primary OP is the most prevalent, and develops naturally during the natural process of aging, especially in connection with menopause and hormonal changes. It includes type I (postmenopausal) and type II (age-related) OP [63]. In individuals with inadequate bone density, secondary OP makes up a sizable number of cases and is frequently disregarded. Secondary OP may affect up to 30% of postmenopausal women, more than 50% of premenopausal women, and 50% to 80% of males [62]. Secondary OP can be caused by numerous factors, including systemic diseases (inflammatory bowel disease, rheumatoid arthritis), endocrine disorders (Cushing’s syndrome, hyperparathyroidism), malignant neoplasms, and certain medications (glucocorticoids) [64,65]. In addition to this, some other important risk factors include weight, smoking, alcohol usage [66], inactivity, dietary calcium insufficiency, and long-term glucocorticoid use. Non-modifiable factors include gender, age, race, and genetics. Regarding gender, these characteristics may also be more prevalent [67].

The risk is often divided into three categories: low, moderate, and severe. Patients who have confirmed OP, show fracture rates ranging from moderate to high, or who present an osteoporotic fracture, frequently meet the requirements for pharmacologic treatment [68]. Pharmacologic osteoporotic drugs are considered for higher-risk individuals. Their aim is to increase bone density and prevent fractures. Pharmacologic treatments for OP include antiresorptive pharmaceuticals, anabolic (bone-strengthening) medications, and drugs that perform both functions [69,70].

The main cause of OP onset is the alteration of the balance between the activity of the OCs and OB during the bone remodeling process, resulting in increased OC-mediated bone resorption compared to OB-mediated bone formation [71].

The pathogenesis of OP is multifactorial, indeed there are several factors and several processes that contribute to the onset of OP.

**Figure 3:**
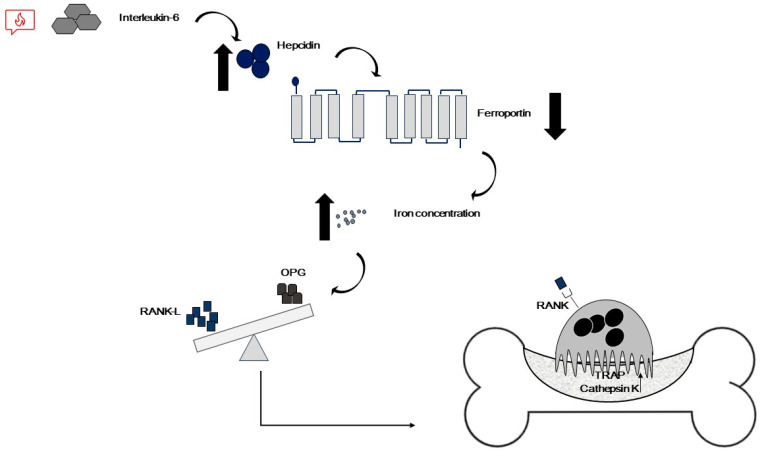
Inflammation alters RANK-L/OPG ratio. Inflammation determines an increase in pro-inflammatory cytokines levels, which leads to an increase in the ratio between RANK-L and OPG, and, consequently, an increase in bone resorption.

Also, iron is a crucial factor implicated in the OP onset; indeed, an excess of iron is responsible for the impairment of bone structure since it is able to alter the process of bone remodeling, promoting resorption and consequently, bone loss [71,72,73]. In particular, an excess of iron inhibits bone mineralization and osteoid maturation [18]. The main cause of iron overload is represented by chronic blood transfusions necessary for different hemoglobinopathies [15,18,73]. Considering that iron metabolism is closely related to inflammation, iron excess could also depend on inflammation. Indeed, increased levels of pro-inflammatory cytokines (in particular IL-6) determines a strong release and activation of hepcidin which is responsible for ferroportin degradation and, consequently, iron accumulation inside the cells. Intracellular iron excess contributes to an increase in the inflammatory state, by determining the production of reactive species of oxygen and nitrogen (ROS and RNS) [74,75]. Elevated levels of pro-inflammatory cytokines determine an increase in the ratio between RANK-L and OPG, thus resulting in bone resorption [71] (Figure 3).

## 3. Bone Metabolism Alteration in Hemoglobinopathies

OP is a very frequent and hard to treat complication in patients affected by hemoglobinopathies. The OP pathogenesis associated with hemoglobinopathies is multifactorial and a general linkage among anemia and risk of OP has recently emerged in the literature [76]. The two most prevalent groups of hemoglobinopathies, thalassemia and sickle cells disorders, have been extensively investigated for bone abnormalities [77].

There are several biomarkers reported to have a significative association with TM or SCD (Table 1). These biomarkers show a low association with sex, and, usually, tend to emerge during childhood, although, apart from supplementations, no specific therapies are approved in this patient population (Table 1).

### 3.1. Bone Metabolism Alteration in Thalassemic Patients

Rise in life expectancy due to improved therapies in TM patients is complicated by the occurrence of associated systemic comorbidities, mainly OP and fractures [104]. Thalassemic patients may experience several bone abnormalities, which are complicated illnesses brought on by several variables that impair the developing skeleton [105]. Prevalence of OP in TM ranges roughly from 51 to 74 percent in lumbar spine and approximately 11 to 38 percent in femur [11,98,106,107]. Patients with thalassemia start to develop bone diseases early in life, since adolescence. This is a crucial period for bone accretion and many factors described below, directly and/or indirectly, affect osteoblastic population, leading to decreased bone formation, while others often increase osteoclastic bone resorption. Pathogenesis is unfortunately still not fully clarified [28]. Several studies have shown that multiple factors play a role in producing bone alterations in TM (i.e., bone marrow expansion, hypogonadism, defective GH–IGF-1 axis, altered cytokines levels, bone iron deposit, deferoxamine toxicity, genetic disorders, and vitamin D and zinc deficiency) [98]. These factors interact and contribute to an unbalanced bone turnover along with higher OCs activity and lower OBs function causing decreased bone mass [11,108,109,110].

The defective bone turnover plays a key role in the low bone mass in thalassemic patients. Elevated bone turnover in adults with thalassemia does not permit constructive bone accrual and attainment of optimal peak bone mass.

#### 3.1.1. TM-Associated OP: Involved Cells and Pathways

The pathophysiology of bone disease in thalassemia is mostly caused by inadequate erythropoiesis, which has led to bone marrow hyperplasia in the past, before transfusions [28].

*Bone marrow enlargement is indeed a common feature of this disease, and it is associated with hepatosplenomegaly, OP, and abnormalities of the bones because of an accumulation of erythroid precursors in the spleen and liver* [111]. Several other factors involved in the pathogenesis of OP related to TM are bone marrow expansion, endocrine complication disfunction, iron overload due to blood transfusion, iron chelation therapy, endocrine complications (chiefly hypogonadism), genetic disorder, renal dysfunction, and deficiency of vitamin D and zinc. These factors interact and contribute to an unbalanced bone turnover along with higher OCs activity and lower OBs function, causing decreased bone mass [11,108,109,110].

The mechanism involved in reduced bone mass in thalassemic patients is still not well understood and it could be due to altered bone turnover. To this extent, several reports link PTH deregulated function with OP onset, or worse, with hypopharathyroidism, which is considered to be one of the most common co-morbidities in this patient population [4]. An increase in bone resorption is demonstrated by elevated levels of urinary bone resorption markers. Several investigations examining indicators of bone resorption, such as urine levels of N-telopeptide, collagen type 1, blood levels of tartrate resistant acid phosphatase isoform-5b, and urinary pyridinium cross-links, have revealed higher OCs activation in these subjects. The altered cytokine network frequently seen in these individuals may be the mechanism behind the OCs activation in well-treated thalassemic patients [98]. Bone resorption can also be a consequence of marrow hyperplasia which leads to the release of cytokines, like IL-6, which in turn increase the oxidative stress and stimulate OC activity. Some studies have shown an elevated ratio of RANK-L/OPG which is a confirmation of high turnover bone disease.

Thalassemic patients often suffer from hypogonadism; therefore, bone enhanced resorption in thalassemia is somewhat due to this endocrine complication [11,105,112]. In TM patients, multiple endocrine dysfunctions—including hypogonadism, growth failure, hyperglycemia, hypothyroidism, and hypoparathyroidism—are reported. There is some evidence that attests to a link between these anomalies and iron overload [98]. Iron overload has been reported as a common cause of increased bone resorption and decreased bone formation [113] and, therefore, chelating agents impose a positive impact on bone metabolism as they reduce this effect [114]. However, improper utilization of iron chelators, high dose administration or incomplete evaluation of patients may develop several negative effects such as platyspondyly, arthropathy, and pathological growth of long bones, probably due to the disrupted OPG/RANK/RANK-L mechanism. The disrupted OPG/RANK/RANK-L pathway functioning leads to increased activity of OCs and decreased proliferation of OBs and fibroblasts, as well as an insufficient amount of minerals and trace elements, like calcium, phosphorus, zinc, and vitamin D in the bone [19,31,115,116,117,118,119,120].

Recently, it has been proposed that the Wnt/β-catenin canonical pathway plays a crucial role in bone remodeling by encouraging OBs precursor cell proliferation and differentiation, decreasing mature OBs apoptosis, and promoting the capacity of differentiated OBs to inhibit OCs differentiation. The negative regulation of this mechanism is involved in the etiology of OP in TM patients. In particular, both DICKKOPF-1 and sclerostin seem to be negative regulators of this system [98].

#### 3.1.2. TM-Associated OP: Genetic Factors

**Figure 4:**
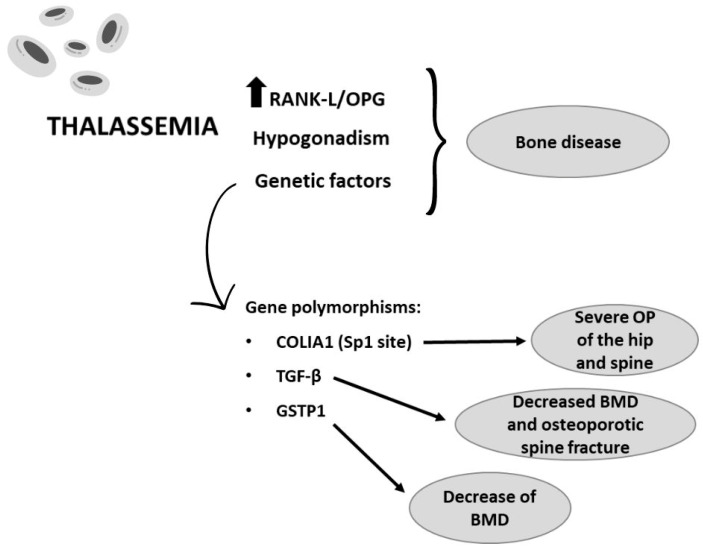
Bone metabolism alteration in thalassemia. The alteration of bone metabolism in TM patients is due to both an increase in RANK-L/OPG ratio and polymorphisms of different genetic factors responsible for osteoporosis (OP) and loss of bone mass density (BMD).

Genetic factors also are involved in TM-associated OP. Several gene polymorphisms have been reported to play a pivotal role in BMD, for example, gene variants in collagen type I A1 (COLIA 1), TGF-beta, and polymorphism of vitamin D receptor (VDR) [121,122]. Wonke et al. reported that 30% of thalassemic patients were heterozygotes (Ss) for the Sp1 site of the COLIA1 gene, while 4% tested homozygotes (SS). More severe OP of the hip and spine was associated with the Sp1 variant in male in comparison to female patients. It is also observed that the spinal bone density decrease in male patients with the Sp1 mutation exhibits a failure of improvement with bisphosphonate therapy [121]. In humans, the TGF-β1 encoding gene, the most ample growth factor in bone, is the principal candidate for the bone density genetic regulation [123]. OBs generate it to inhibit the proliferation and activity of OCs and activate proliferation of pre-OBs. Several reported polymorphisms of TGF-β1 have been involved in decreased BMD, allowing for osteoporotic spine fractures. The rank order which leads to increased fracture prevalence of TGF- β1 genotypes is CC < CT < TT; additionally, the Cc genotype in TM patients was observed as a factor leading to low bone density [122,123,124]. Elevated oxidative stress and the genes controlling it are also correlated to OP. Ile105 Val—a functional polymorphism of glutathione S-transferase P1 (GSTP1)—reduces the anti-oxidative property of GSTP1, and it is reported to lead to a decrease in BMD in TM patients [125] (Figure 4).

### 3.2. Bone Metabolism Alteration in Sickle Cell Patients

Sickle cell related disorders are reported to manifest an alteration of bone metabolism which is responsible for the onset and development of fractures, chronic pain, and long-term disability [126]. In this category of patients, low bone mineral density is detected at an earlier age than in the normal population. Many risk factors, such as vitamin D deficiency, hemolysis, inflammation, impaired diet/malnutrition, and hypogonadism are common among different anemia/hemoglobinopathies [127]. The main disease-specific causes of bone impairment in SCD patients are, instead, the avascular necrosis events, which affect mostly femoral heads, and also the acute vaso-occlusive crisis (VOC), which is responsible for increased inflammation and tissue ischemic damage [38,39].

VOC crisis, together with the chronic consequences of anemia, affect the musculoskeletal system, influencing several processes, such as marrow hyperplasia, osteonecrosis, extramedullary hematopoiesis, osteomyelitis, myonecrosis, and reversion of yellow marrow to red marrow [128,129].

In SCD, several metabolic and endocrinological alterations are described, mainly due to the effects of VOCs; more rarely, endocrinological alterations are due to complications of chronic transfusion therapy (iron deposits), similar to what is observed in thalassemia. It is reported that 80% of SCD young adult patients show a decrease in BMD, OP, osteopenia, vertebral collapse, increased risk of fractures, and bone pain [130,131,132]. As anticipated above, several factors can influence bone loss in these patients, for example low physical activity, malnutrition, high levels of inflammatory cytokines and a reduction in vitamin D concentration, hemolysis, inflammation, underweight, and hypogonadism [39]. In SCD, a deficiency of zinc, folic acid and vitamins—especially vitamins A, B6, B12, C, E and D—is observed in the majority of patients. Compared with healthy subjects, SCD children showed a poor nutritional status, an alteration of growth, and postponed puberty [133].

Low bone mass density (BMD) is observed both in pediatric and adult SCD patients [134,135,136]. Approximately 70–80% of adults show low BMD and approximately 13% are classified as OP patients [137]. The lumbar spine appears to be particularly susceptible to OP changes [138]. The genesis of osteopenia is multifactorial and is linked to the nutritional status (low BMI, low blood concentrations of zinc, low hemoglobin levels, nutritional deficiencies of calcium, magnesium and vitamin D), to the hormonal status (male sex, delayed puberty, low levels of estradiol, testosterone, and PTH), and the metabolic state (bone microinfarctions due to repeated sickle cell crises) [83,92,138,139].

**Figure 5:**
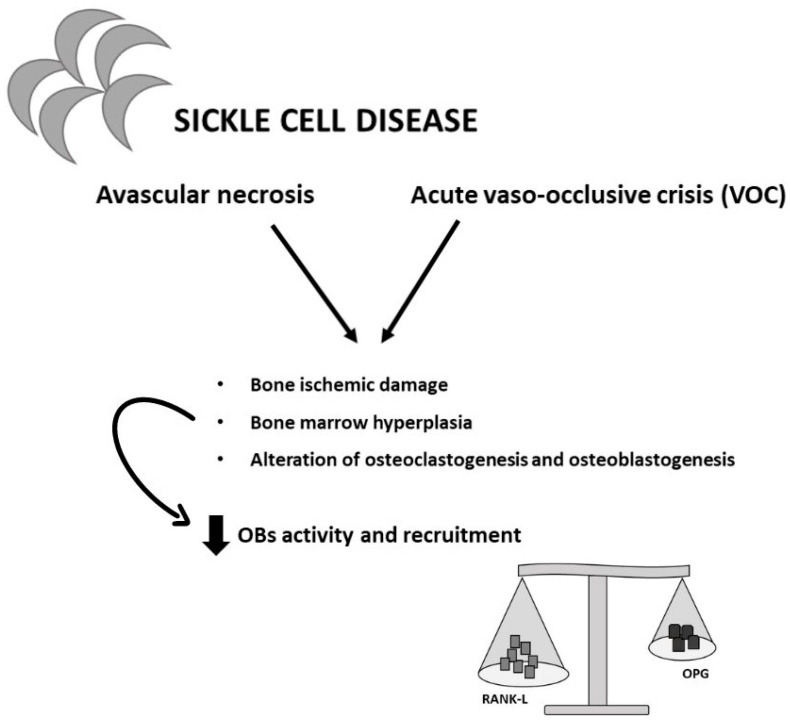
Bone alteration in sickle cell disease. SCD patients experience both avascular necrosis and acute vaso-occlusive crisis (VOC). These processes lead to an inflammatory state. This results in an increase in RANK-L and a reduction in osteoblasts (OBs) activity and maturation, resulting in a loss of bone mass density.

However, there is not a lot of knowledge about the main processes responsible for the onset of sickle bone disease (SBD) and there is no specific therapy to counteract bone metabolism alteration in SCD patients. In SCD, VOC onset causes bone ischemic damage and a hyperplasy of bone marrow which inhibit OBs recruitment. Recurrent infarcts of bone and bone marrow (BM) in individuals with SCD are a contributing factor to localized oxygen and nutrition shortages [38]. Indeed, SCD patients show an impairment of the equilibrium between osteoclastogenesis and osteoblastogenesis with a decrease in OBs activity and recruitment. In particular, as well as in thalassemia, the impairment of the RANK/RANKL/ OPG axis—the main responsible factor for osteoclastogenesis regulation—seems to be involved in the alteration of bone metabolism [38,39,116] (Figure 5).

SCD patients have an higher degree of hemolysis linked to the onset of bone impairment with mechanisms independent of erythropoietic stress [140]. Moreover, the increase in incidences of very low levels of vitamin D and sex hormones—in particular estradiol—in SCD patients are involved in the alteration of bone metabolism [39,40]. Various factors have been proposed to explain the high prevalence of vitamin D deficiency in SCD patients, including the dark skin type that affects 50% of our patients, the reduced intestinal absorption of vitamin D, likely due to chronic cholestasis, and higher levels of body fat tissue in SCD patients may further induce vitamin D deficiency by sequestration, hence explaining its low bioavailability [38].

Children with SCD frequently have pubertal delay and development failure and significantly frequent endocrine problems, including those related to low-grade inflammatory illnesses. The growth hormone (GH)–IGF-1–IGFBP3 axis has indeed been shown to be impaired in SCD patients. Therefore, hypothyroidism, hypogonadism, insulin resistance, and growth hormone insufficiency are frequent features of SCD [39].

## 4. Therapeutic Intervention and Novel Evidence for the Treatment of Osteoporosis in Patients with Hemoglobinopathies

OP management in patients with hemoglobinopathies requires a multidisciplinary approach. Controlling anemia, appropriate chelation therapy, a balanced diet and lifestyle, consistent exercise, proper management of coexisting illnesses, hormone replacement therapy for people with hypogonadism, and vitamin D and calcium therapy or supplementation are just few examples of general interventions [141].

Several studies have focused on the effect of iron accumulation in bone in transfusion-dependent patients which causes focal osteomalacia, inhibiting osteoid development and mineralization, and decreasing the tensile strength of the bone unit [18]. TM patients with high ferritin and liver iron concentration (LIC) levels exhibit increased OCs activity, as confirmed by high levels of TRAP in OCs.

Chelation therapy is therefore able to restore OCs activity, bringing it back to its normal physiological balance, representing a good treatment for TM-related osteopenia and OP. Deferoxamine (DFO), deferiprone (DFP), and deferasirox (DFX), three common iron chelators, have been authorized for clinical usage [18,28].

Baseline laboratory testing for proper OP prevention should involve the assessment of serum calcium, phosphate, creatinine, albumin (for the correction of serum calcium), parathyroid hormone (PTH), 25-hydroxy-vitamin D (25(OH)D), 24 h urinary calcium and phosphate excretion, markers of bone turnover, and laboratory tests to assess gonadal function.

Dual energy X-ray absorptiometry (DEXA), which measures bone mass at the lumbar spine and proximal femur, is the most accurate and trendy way to measure bone mineral density (BMD) [105]. The prevalence of OP in TM is about 10.8–37.9% at the femur site and 50.7–74.1% at the lumbar spine (LS). The frequency of fractures is 12.1–35.1%, occurring mainly at the extremities.

BP represent the most effective therapy in β-thalassemic patients. The first choice of therapy in thalassemic patients with OP is either represented by zoledronic acid administered intravenously at a dose of 4 mg every 3 months or every 6 months, or neridronate intravenously administered at a dose of 100 mg once every three months, both to be regarded off-label (category IA) [28]. Their off-label use is allowed due to their positive effects on the BMD, bone turnover, and pain [28,141] and by their tested effect in the patient population [106]. Other BP can be used as second-line therapy or in specific patient populations; Alendronate is one of the oral BPs which can be administered once a week at a dose of 70 mg. It achieved better results in terms of BMD growth in a randomized controlled trial (RCT) that directly compared it to intramuscular clodronate 100 mg administered every ten days [142]. The administration of a monthly dose of pamidronate 90 mg increases the BMD at level of LS [142]. An increase in BMD at level of LS, forearm, and FN is reported after the administration of BPs and after the addition of zinc sulfate [105,142].

The management of OP in patients with hemoglobinopathies is further complicated by the potential interaction between hemoglobinopathy and OP treatments. For example, patients with SCD show an increased risk of developing jaw osteonecrosis after BP treatment, while iron chelation therapy used in thalassemia patients may lead to osteopenia [143].

Teriparatide (TP) is a recombinant portion of human parathyroid hormone (PTH), constituted by 34 amino acids. It represents the only anabolic therapy approved in Italy for the treatment of widespread OP [28] and it is recommended to treat OP in males and postmenopausal women who present high risk of fracture, also due to prolonged systemic glucocorticoid medication. TP has positive effects in terms of improved BMD in a lot of different hereditary and secondary disorders linked to bone fragility [142].

It is also reported that TP is responsible for increasing the transcriptional expression levels of Insulin-like growth factor (IGF)-1 and fibroblast growth factor 2 (FGF2), two pro-osteoblastogenic growth factors. TP promotes bone formation by directly influencing activity of OBs precursors, mature OBs, bone lining cells, and the Wnt-pathway in OBs and osteocytes, as well as by inhibiting sclerostin via Wnt-ligand independent pathways [142]. 

The number of vertebral and nonvertebral fractures was indeed reduced by 74% and 39%, respectively, in a recent metanalysis that compared TP with placebo. The frequency and severity of back pain were also shown to decrease after TP therapy [28].

Osteoanabolic drugs are more effective than BPs in preventing fractures in patients with high risk of fracture, and current research suggests that the administration of these drugs before treatment with anti-resorptive agents leads to greater increases in BMD. A team of Italian researchers have suggested TP as a second-line therapy in cases of severe TM-associated OP with a lot of fractures following protracted BP medication, in patients without any responses to BP therapy, or in patients with significant side effects after BP administration [28].

Adults with thalassemia-related bone damage may benefit from TP, particularly if they require long-term, sequential therapy [105].

Another drug sporadically used in treating OP in hemoglobinopathies is Denosumab, a monoclonal antibody that binds to RANK-L, inhibiting it. It is indicated for the treatment of OP in postmenopausal women and men who are at increased risk of fractures [141].

In a recently published uncontrolled observational study of Denosumab (60 mg SC every six months), a RANK-L was shown to improve DXA-measured BMD and reduce skeletal turnover in β-thalassemia patients with OP [10]. TM patients after one year of Denosumab treatment show a considerable rise in BMD density, as determined by dual energy X-ray absorption, in the lumbar spine and femoral neck, as well as a quick drop in type 1 collagen carboxy telopeptide levels [105].

The recent evidence highlighting the possibility to target EC/EV system to contain OCs activity in OP related to hemoglobinopathies is exploratory, since human OCs express all the components of this system [26,35]. In particular, the EC/EV system has a key role in the modulation of bone cell activity [34,36,144,145,146,147,148,149,150]. CB2 stimulation is responsible for OCs activity inhibition by contributing to bone mineralization; CB1 and TRPV1 stimulation instead stimulates OCs activity by favoring the process of bone degradation [34,144,145,146,147,148,149,150].

Interestingly, Rossi et al. in 2014 demonstrated that high levels of ferritin and LIC are responsible for the increase in OCs activity, by revealing high levels of TRAP and CTK, responsible for bone matrix degradation. Moreover, they also revealed that the activation or the desensitization of TRPV1 is responsible for the modulation of TRAP expression and activity and that they are also dependent on iron concentration, highlighting once again the involvement of iron overload in bone metabolism alteration in TM patients. In particular, administration of iron chelators decreases TRAP and CTK levels in TM patients-derived OCs, by reducing activity of OCs and decreasing the osteoporotic processes [26]. Therefore, the use of iron chelators has a double positive effect for β-TM patients, counteracting not only iron overload but also reducing osteoclastic activity and therefore preventing OP.

**Figure 6:**
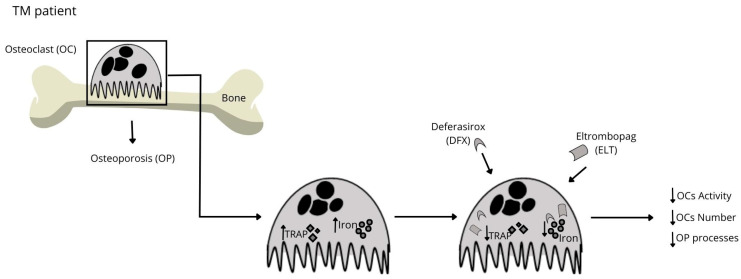
Iron chelators in counteracting bone metabolism impairment in hemoglobinopathies. The chelation therapy based on the employment of different iron chelators as DFX and ELT, is able to restore the normal physiological activity of OCs, reducing/preventing the OP process.

In 2018, Punzo et al. for the first time proposed the use of Eltrombopag (ELT), a thrombopoietin receptor agonist approved for the treatment of chronic immune thrombocytopenia, as an iron chelator also on primary human OCs from thalassemic patients. The administration of ELT not only reduced intracellular iron concentration but also decreased expression of TRAP, and OCs numbers and activity, thus preventing bone mass loss and consequently, osteoporotic mechanisms. These effects were stronger when ELT was administered together with DFX, suggesting a synergism between the two drugs [15] (Figure 6).

There is no evidence regarding a specific treatment to counteract or prevent bone metabolism impairment in SCD subjects. But considering the low levels of vitamin D in these patients, it could be useful to repeat its supplementation [151]. BP can be used also in SCD patients in order to reduce pain and counteract bone impairment. Different studies tested two different BPs in SCD, zoledronic acid and alendronate, to highlight their effect on bone. It was reported that only alendronate had the ability to increase BMD in SCD patients with OP [151]. Certainly, further research and investigations will be necessary in order to better understand the role of BPs in SCD.

## 5. Conclusions

Alteration of bone health represents one of the most severe complications in patients affected by hemoglobinopathies, resulting mainly in bone loss.

Bone metabolism is a finely regulated process, during which there is a balance between bone matrix deposition and bone resorption, and also between the activity of OCs and OBs. Impairment of bone metabolism is responsible for the onset of bone diseases, such as OP.

Hemoglobinopathies have a compound etiopathogenesis profile due, primarily, to the anemic state per se inducing hypoxia with a negative impact on OB recruitment, as well as an increase in systemic cytokines’ patterns, inducing OC precursors functions and bone loss. Iron overload, due to chronic transfusion regimens, could also contribute to the onset of bone complications causing an impairment of bone metabolism, thus promoting resorptive processes, determining an increased activation of OCs. Moreover, a delayed pubertal growth spurt and low peak bone mass, as well as more profound primary or secondary hypogonadisms, may explain the high rate of OP in these categories compared to a healthy population. Vitamin D deficiency, last but not least, can increase bones’ resorptive state.

Specific factors, such as drug toxicities (i.e., iron chelators), bone marrow hyperplasia, and ischemic damage secondary to vaso-occlusive crisis (VOC), as well as alteration of the hepcidin–ferroportin axis in SCD patients, can be behind differences in the features of bones alterations in the different patient populations. On top of all that, there are still unknown genetic and acquired factors playing a role in the onset and severity of bone disease in patients with hemoglobinopathies.

BPs are the main class of drugs used in TM to reduce bone resorption and to increase BMD. Also, the use of TP is suggested to counteract OP in TM patients, since several studies revealed its capability to promote an increase in BMD in TM patients, by promoting the activation of OBs and inhibiting their apoptosis. No clear indications are provided in internation guidelines to treat, counteract and/or prevent SBD in SCD patients; although, supplementation of vitamin D or the use of BP can be useful to improve BMD in SCD.

Recent scientific evidence highlights the possibility to target the EC/EV system in order to counteract OP, based on the fact that stimulation of the CB2 receptor promotes the activation of OBs and the stimulation of CB1 and TRPV1 induced the activation of OCs. In particular, TRPV1 activation in TM patients after treatment with iron chelators reduces the activation of OCs by reducing TRAP expression levels, demonstrating that iron modulates TRPV1 activation and desensitization.

Another novel therapeutic option to counteract iron overload and consequently, OCs activity alteration in TM patients, is represented by ELT, which has been demonstrated to exert iron chelating properties on human OCs derived from TM patients, reducing OCs activity, and preventing OP onset.

In conclusion, the management of bone metabolism impairment in patients with hemoglobinopathies is a complex process that requires a holistic and individualized approach. Careful evaluation of the patient’s hemoglobinopathy and bone health status is necessary to develop an appropriate treatment scheme which considers the potential risks and benefits of therapy.

## Figures and Tables

**Table 1:** Biomarkers associated with TM or SCD. Non comprehensive list of biomarkers reported to have a significative association with TM or SCD. Data shown have been all identified as statistically significant in the referenced papers: a: adults; c: children; TM: thalassemia major; SCD: sickle cell disease; B/S: beta-thalassemia/HbS; NA: not applicable; UN: unavailable; H: healthy subjects; P: patients; * Review.

Marker	Change	Disease	Values	References
**Urinary Deoxypyridinoline (D-PYR)**	Increase	TM	(pmol/μmol ur.creat.) aH: 14.8 ± 5.6 aP: 27.8 ± 8.7	Morabito, 2007 [78]
		SCD	(nM/mM) aH: 5.4 ± 2.7 aP: 11.1 ± 4.4	Bolarin, 2010 [79]
**Osteocalcin**	Decrease	TM	(pmol/mL) aH: 5.6 ± 1.9 aP: 2.4 ± 1.1 (ng/mL) aH: 5.6 ± 1.9 aP: 2.4 ± 1.1 cH: 13.69 ± 0.92 cP: 5.68 ± 3.22	Morabito, 2007 [78] Morabito, 2004 [24] Abd El-Moneim, 2018 [80]
		SCD	(ng/mL) aH: 12.57 (2.48–30.19) aP: 10.93 (1.04–43.24) (ng/mul) aP: 12.3 ± 3.7	Voskaridou, 2005 [81] Adewoye, 2008 [82]
**Tartrate-resistant acid phosphatase 5b**	Increase	TM	(U/L) aH: 0.91 ± 0.08 aP: 1.01 ± 0.32	Abd El-Moneim, 2018 [80]
		SCD	aH: 2.4 (2.0–2.9) aP: 4.4 (3.3–5.7)	Nouraie, 2011 [83]
**Alkaline phosphatase**	Increase	TM	(µg/L) a,cH: 171.3 ± 41.7 a,cP: 194.1 ± 37.1	Salama, 2006 [84]
		SCD	(U/L) aH: 20.99 (9.45–27.51) aP: 32.83 (17.25–61.43) aH: 41.33 ± 12.4 aP: 85.60 ± 33.3	Voskaridou, 2005 [81] Fung, 2008 [85]
**sRANKL/OPG**	Increase	TM	aH: 1.25 ± 0.2 aP: 2.50 ± 0.2 a,cH: 0.9 a,cP: 1.38 cH: 33.24 cP: 46.09	Morabito, 2004 [24] Angelopulos, 2007 [86] Celik, 2022 [87]
**sRANK**	Increase	TM	(pmol/L) aH: 4.5 ± 1.2 aP: 8.1 ± 2.8	Morabito, 2004 [24] Morabito, 2007 [78]
**OPG**	Decrease	TM	(pmol/L) cH: 2.43 ± 0.57 cP: 2.01 ± 0.66	Celik, 2022 [87]
**Osteoclastic Markers**	Increase	TM	a,cNA	Salama, 2006 [84] Toumba, 2010 [88]
**Amino-terminal pro-peptide of type I procollagen (P1NP)**	Decrease	TM	aUN	Baldini, 2014 [89]
**C-telopeptide of type-I collagen**	Decrease	B/S	(ng/mL) aH: 45.98 (5.04–124) aP: 135.73 (39.93–246.1)	Voskaridou, 2005 [81]
**PTH**	Decrease	TM	(µg/mL) cH: 75.2 ± 31.3 cP: 35.3 ± 15.2 cH: 44.3 ± 5.63 cP: 32.2 ± 0.96	Pirinççioğlu, 2017 [90] Goyal, 2010 [91]
		SCD	(pmol/L) aH: 4.11 ± 1.46 aP: 2.22 ± 1.26	Elshal, 2012 [92]
**Vitamin D**	Decrease	TM/SCD	a,cNA	Manolopoulos, 2021 * [93] Soe, 2017 * [94]
**Serum IGF1**	Decrease	TM	(nmol/mL) aH: 35.25 ± 8.33 aP: 21.07 ± 5.12 (ng/mL) aH: 185.8 ± 27.7 aP: 55.8 ± 16.0	Giordano, 2021 * [39] Lasco, 2002 [95] Morabito, 2004 [24]
		SCD	(nmol/mL) cH: 42.88 ± 4.33 cP: 18.09 ± 3.88	Luporini, 2001 [96]
**IGFBP-3**	Decrease	TM	(mg/mL) aH: 2.5 ± 0.1 aP: 1.9 ± 0.4	Lasco, 2002 [95]
**Sexual Hormones (i.e., Estradiol)**	Decrease	TM	(pg/mL) aH: 90.8 (62.2–136.1) aP: 13.8 (5.0–45.7)	Anapliotou, 1995 [97] Gaudio, 2019 * [98] Thavonlun, 2023 [99]
**IL-6**	Increase	TM	(pg/mL) aH: 6.2 ± 3.7 aP: 8.1 ± 3.3	Morabito, 2007 [78] Gaudio, 2019 * [98]
**IL-1α**	Increase	TM	(pg/mL) aH: 5.6 ± 2.5 aP:13.2 ± 4.1	Morabito, 2007 [78] Gaudio, 2019 * [98]
**TNF-α**	Increase	TM	(pg/mL) aH: 6.4 ± 2.1 aP:11.4 ± 5.3	Morabito, 2007 [78]
**GDF15**	Increase	TM	aUN	Teawtrakul, 2023 [100]
**Klotho**	Decrease	TM	(pg/mL) aH: 618.2 ± 141.1 aP: 558.7 ± 160.5	Baldan, 2015 [101]
**Sclerostin**	Increase	TM	(pg/mL) aH: 250 (0–720) aP: 605 (22–1227)	Voskaridou, 2012 [102]
**Arachidonic Acid**	Increase	TM	(nM) aH: 8 aP: 52	Piriyakhuntorn, 2023 [103]
**Glutamate**	Increase	TM	(µM) aH: 8.3 aP: 24.8	Piriyakhuntorn, 2023 [103]
**Glutamine**	Decrease	TM	(AU) aH: 11.4 aP: 10.0	Piriyakhuntorn, 2023 [103]

## Data Availability

No new data were created or analyzed in this study.
